# Economic evaluation of population-based type 2 diabetes mellitus screening at different healthcare settings in Vietnam

**DOI:** 10.1371/journal.pone.0261231

**Published:** 2021-12-23

**Authors:** Phung Lam Toi, Olivia Wu, Montarat Thavorncharoensap, Varalak Srinonprasert, Thunyarat Anothaisintawee, Ammarin Thakkinstian, Nguyen Khanh Phuong, Usa Chaikledkaew

**Affiliations:** 1 Mahidol University Health Technology Assessment (MUHTA) Graduate Program, Mahidol University, Bangkok, Thailand; 2 Health Strategy and Policy Institute, Ministry of Health, Hanoi, Vietnam; 3 Health Economics and Health Technology Assessment (HEHTA), Institute of Health and Wellbeing, University of Glasgow, Glasgow, United Kingdom; 4 Faculty of Pharmacy, Social and Administrative Pharmacy Division, Department of Pharmacy, Mahidol University, Bangkok, Thailand; 5 Faculty of Medicine Siriraj Hospital, Health Policy Unit, Mahidol University, Bangkok, Thailand; 6 Faculty of Medicine Ramathibodi Hospital, Department of Family Medicine, Mahidol University, Bangkok, Thailand; 7 Faculty of Medicine Ramathibodi Hospital, Department of Clinical Epidemiology and Biostatistics, Mahidol University, Bangkok, Thailand; Universidad Nacional Autonoma de Nicaragua Leon, NICARAGUA

## Abstract

**Introduction:**

Few economic evaluations have assessed the cost-effectiveness of screening type-2 diabetes mellitus (T2DM) in different healthcare settings. This study aims to evaluate the value for money of various T2DM screening strategies in Vietnam.

**Methods:**

A decision analytical model was constructed to compare costs and quality-adjusted life years (QALYs) of T2DM screening in different health care settings, including (1) screening at commune health station (CHS) and (2) screening at district health center (DHC), with no screening as the current practice. We further explored the costs and QALYs of different initial screening ages and different screening intervals. Cost and utility data were obtained by primary data collection in Vietnam. Incremental cost-effectiveness ratios were calculated from societal and payer perspectives, while uncertainty analysis was performed to explore parameter uncertainties.

**Results:**

Annual T2DM screening at either CHS or DHC was cost-effective in Vietnam, from both societal and payer perspectives. Annual screening at CHS was found as the best screening strategy in terms of value for money. From a societal perspective, annual screening at CHS from initial age of 40 years was associated with 0.40 QALYs gained while saving US$ 186.21. Meanwhile, one-off screening was not cost-effective when screening for people younger than 35 years old at both CHS and DHC.

**Conclusions:**

T2DM screening should be included in the Vietnamese health benefits package, and annual screening at either CHS or DHC is recommended.

## Introduction

Globally, the number of people living with diabetes has been estimated to increase from 463 million in 2019 to 578 million in 2030, with the number estimated to increase by 51% (700 million) in 2045 [1]. Around 90% of diabetes cases are type 2 diabetes mellitus (T2DM) which results in substantial morbidity and mortality for those afflicted, and is correlated with a high cost for health care [2]. In Vietnam, the prevalence of T2DM stood at 5.4% in 2012 [3], which is relatively low compared to neighboring countries. However, local evidence suggests that there is a rising trend in the incidence of disease, despite various public health interventions already implemented [4,5]. Furthermore, recent estimates showed that the total direct medical costs among Vietnamese people with diabetes were US$ 435 million in 2017 [6]. It will be a significant economic burden to the country if additional efforts are not made.

The American Diabetes Association (ADA) recommends screening for T2DM among people at risk and also can be started in all individuals without risk who aged 45 years onwards [7]. Routine screening of T2DM patients will allow early management for those who are diagnosed at an early stage of T2DM and can help prevent the burden of complications and mortality [8]. Many screening tests i.e., fasting plasma glucose (FPG) test, hemoglobin A1C (HbA1c), and oral glucose tolerance (OGTT) are currently recommended [7]. However, the implementation of these tests might be difficult in some circumstances, as they require standardized laboratory facilities and more health resources. The Vietnamese guideline on T2DM’s diagnosis and treatment also follows the ADA’s guideline suggesting that T2DM screening should be performed in those who at risk or those who aged ≥ 45 irrespective of their risk status. Nevertheless, in Vietnam, one of limited health resources setting, fasting capillary glucose (FCG) test [9] and FPG [10] were found to be appropriate and recommended for diabetic screening tests with the combination of these tests will result in higher accuracy [11].

Previous systematic review evidence suggests that screening for T2DM is value for money in many settings compared to no screening [12]. However, variations regarding screening tests and methodology used among previous economic evaluations have been found [12], and this restricts the generalizability and transferability of the results. Moreover, almost all studies [13–17] did not consider how to implement the screening program in healthcare system context.

At present, the Vietnamese Ministry of Health has currently set the goal to identify at least 40% undetected individuals with diabetes [18]. According to current guidance on diabetic screening tests, FCG test is provided at the commune health station (CHS) to identify suspected patients and confirm diabetes diagnosis by FPG test at district health centers (DHC), whereas FPG test is initially performed at DHC. Nevertheless, there has been no economic evaluation information on which screening tests for T2DM should be adopted in the Vietnamese health benefit packages reimbursed by the Health Insurance Scheme. Therefore, this study aimed to compare costs and health outcomes of screening for T2DM at CHS and DHC with different screening intervals (i.e., one-off screening, annual screening, and 3-yearly screening) and different initial age of screen for T2DM in Vietnam. The results of this study will serve as the evidence for policymakers and stakeholders on development of screening policy as well as health benefit package in the country. Moreover, this study can contribute to global knowledge on the cost-effectiveness of T2DM screening services when implemented at different healthcare levels, especially in low- and middle-income country context where has limited healthcare budget and resources.

## Materials and methods

### Target population

Cost-utility analysis was done through a hybrid of decision tree and Markov models to estimate lifetime costs and outcomes of different screening strategies based on a governmental and societal perspectives. A hypothetical cohort of people aged 40 years was used as the base case for simulating the natural history model of T2DM. Individuals who have already been diagnosed with T2DM were excluded from the screening program.

### Intervention and comparator

This study compared no screening i.e., current practice in Vietnam with two proposed screening strategies integrated into primary healthcare facilities: (1) screening at CHS and (2) screening at DHC. We also compared costs and health outcomes on different starting ages for screening at 30, 35, 40, and 45 years as well as different screening intervals i.e., one-off, annual, and 3-yearly screening. For the screening scenario on screening at CHS, participants would go to CHS for T2DM screening. Initially, risk stratification was carried out by the Finnish Diabetes risk score (FINDRISC) questionnaire in which body mass index (BMI) and waist circumference benchmarks were adjusted for the Asian population. Then, individuals at risk of T2DM were screened by FCG test, and those with positive results were referred to DHC for confirmation test by FPG test. In this case, participants would go to DHC by themselves which corresponded to travel costs as well as the adherence rate of participants. For the screening scenario at DHC, participants would go to DHC for T2DM screening. Risk stratification was also carried out by the FINDRISC questionnaire. However, people who fell into the “at-risk” group would receive an FPG test. If he or she had a positive result (i.e., FPG ≥7 mmol/l), another FPG test would be performed for confirmation.

### Model structure

**Fig 1:**
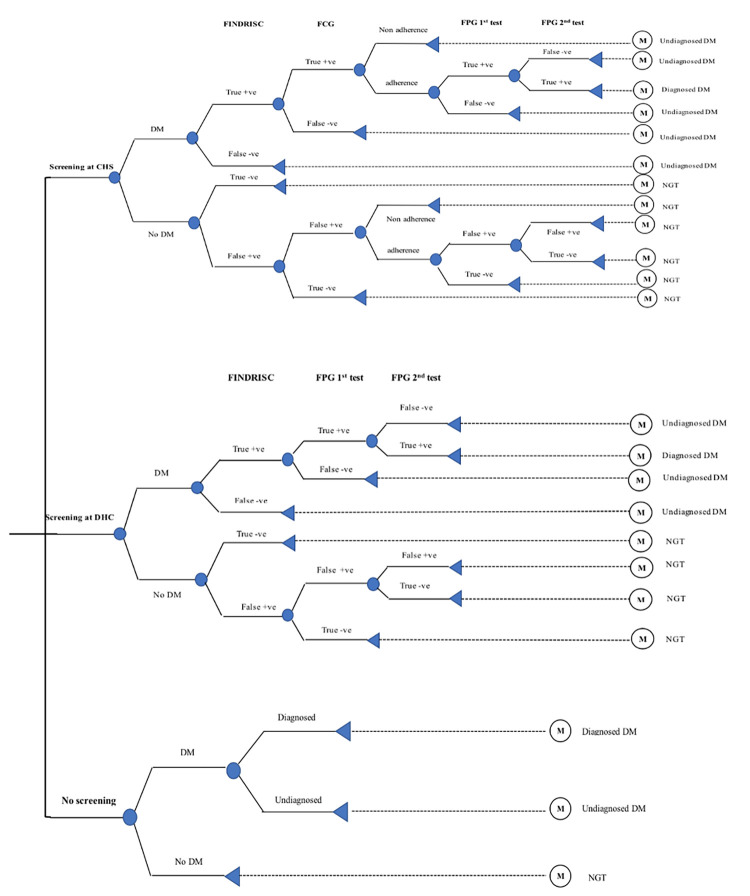
The decision tree.

The hybrid of decision tree and Markov models was constructed and validated by clinical experts in Vietnam. The decision tree represents two proposed screening options i.e., screening at CHS and screening at DHC compared to current practice (i.e., no screening) (Fig 1). A hypothetical cohort of individuals who were free with complications (i.e., coronary heart diseases and kidney diseases) and comorbidities such as hypertension were simulated for the benefits for screening program. In the option of screening at CHS, individuals would be first screened by the FINDRISC. Those who had positive results (i.e., true and false positive) would be screened by FCG tests. If the test results were positive, they would be referred to DHC for confirmation by FPG test. At DHC, if they had two consecutive positive results by FPG (i.e., FPG ≥7 mmol/l), they would be diagnosed as having T2DM according to Vietnamese guidelines [10]. For the screening at DHC, individuals would also be screened by the FRINDRISC at first. If the results were positive, they would be screened by FPG immediately. A second FPG test would be taken place immediately in order to confirm diabetes diagnosis. However, for those who had false positive with FPG twice, we assumed that they would be classified as normal glucose tolerance (NGT), as they would not be treated because of very low possibility for receiving life-long treatment. In current practice (no screening), patients with T2DM would be diagnosed or remain undiagnosed.

**Fig 2:**
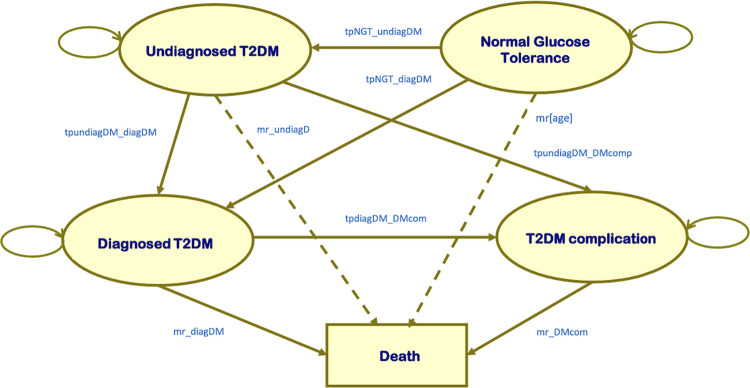
The Markov model.

At the end of each decision tree, the simulated cohort would move to the Markov model (Fig 2) consisting of NGT, undiagnosed T2DM, diagnosed T2DM, T2DM complication, and death states. For the patients diagnosed with T2DM, we assumed that they had no complications. Individuals could remain in the same health state, progress to other health states, or die from all-cause or disease-specific mortality. Costs and health outcomes were estimated in a lifetime horizon with 1-year cycle length using Microsoft Office Excel 2019 (Microsoft Corp., Redmond, WA). Within cycle correction was performed by Simpson’s 1/3 rule correction method proposed by Elbasha and Chhatwal [19].

### Model parameters

Model’s input parameters are presented in Table 1.

**Table 1:** Model’s input parameters.

Input parameter		Mean	Standard Error	Distribution	Source
**Epidemiological data**					
Prevalence of T2DM from 30+		0.0631	0.00002	Beta	[20]
Prevalence of T2DM from 35+		0.0683	0.00003	Beta	[20]
Prevalence of T2DM from 40+		0.0743	0.00004	Beta	[20]
Prevalence of T2DM from 45+		0.0814	0.00005	Beta	[20]
**Screening tests’ performance**					
Sensitivity of FINDRISC		0.864	0.082	Beta	[21]
Specificity of FINDRISC		0.583	0.031	Beta	[21]
Sensitivity of FCG		0.842	0.003	Beta	[9]
Specificity of FCG		0.766	0.001	Beta	[9]
Sensitivity of FPG		0.544	0.006	Beta	[22]
Specificity of FPG		0.989	0.0004	Beta	[22]
**Clinical data**					
HbA1c of undiagnosed T2DM		8.5	2.5	Normal	[23]
HbA1c of diagnosed T2DM		7.0	1.5	Normal	[24]
HbA1c of T2DM complication		9.1	2.4	Normal	[25]
Increased risk of death with T2DM		1.89	0.077	Lognormal	[26]
Mortality multipliers for each 1% increase in HbA1c		1.38	0.048	Lognormal	[27]
Increased risk of complication for each 1% increase in HbA1c		1.40	0.036	Lognormal	[27]
**Transition probability**					
From	To				
NGT	Undiagnosed T2DM	0.0067	0.0011	Beta	[28]
NGT	Diagnosed T2DM	0.0067	0.0011	Beta	[28]
Undiagnosed T2DM	Diagnosed T2DM	0.0352	0.0102	Beta	[29]
Undiagnosed T2DM	T2DM complication	0.0231	0.000001	Beta	Estimated
Diagnosed T2DM	T2DM complication	0.0140	0.000001	Beta	[30]
**Cost (US dollar, 2019)**					
**Direct medical cost**					
*Cost of screening test*					
Screening by FINDRISC		0.3			Estimated
Screening by FCG		0.7			[31]
Screening by FPG		0.9			[31]
*Cost of treatment*					
Treatment for diagnosed T2DM		66.0	76,945	Gamma	Primary data
Treatment for undiagnosed T2DM		17.9			Assumed
Treatment for T2DM complication		160.1	86,476	Gamma	Primary data
**Direct non-medical cost**					
*Direct non-medical cost of screening*					
Screening at CHS		0.6			
Screening at DHC		1.96			
*Direct non-medical cost of treatment*					
Treatment for diagnosed T2DM		228.1	684,856	Gamma	Primary data
Treatment for undiagnosed T2DM		99.6			Assumed
Treatment for T2DM complication		327.1	189,641	Gamma	Primary data
**Utility value**					
NGT		0.91	0.0038	Beta	[32]
Undiagnosed T2DM		0.91	0.0038	Beta	Assumed
Diagnosed T2DM		0.89	0.02	Beta	Primary data
T2DM complication		0.63	0.02	Beta	Primary data

CHS, commune health station; DHC, district health center; FCG, fasting capillary glucose; FPG, fasting plasma glucose; NGT, normal glucose tolerance; T2DM, type-2 diabetes mellitus.

#### Transition probabilities

Transition probabilities from NGT to undiagnosed T2DM and diagnosed T2DM were obtained from a study in China [28] owing to the unavailability of such data in Vietnam. Furthermore, we assumed that transition probability from NGT to undiagnosed T2DM and from NGT to diagnosed T2DM were similar. Transition probability from undiagnosed T2DM to diagnosed T2DM was estimated from a study by Harris et al [29]. Transition probability from diagnosed T2DM to T2DM complications was retrieved from a follow-up study in Taiwan [30]. Transition probability from undiagnosed T2DM to T2DM complication was estimated based on the difference between the predicted HbA1c concentration between undiagnosed T2DM and diagnosed T2DM. Similarly, mortality rates for diabetic states (i.e., undiagnosed T2DM, diagnosed T2DM, and T2DM complication) were adjusted further depending on the predicted difference in HbA1c among health states. The HbA1c level of diagnosed T2DM was obtained from ADDITION-Europe usual care arm with the HbA1c level of 7.0*%* [24]. For undiagnosed T2DM state, we applied the HbA1c level of 8.5*%* among those diagnosed clinically with T2DM within the past 6 months [23]. For diabetic complications, the mean HbA1c level of 9.1*%* was derived from a study in China [25]. All-cause mortality rates were taken from the life-table by the Vietnamese General Statistics Office (GSO) 2019 [33].

#### Costs

According to a social perspective, direct medical and non-medical costs were included, whereas only direct medical costs were incorporated based on a provider perspective. Costs of screening tests were estimated based on activities in the screening service. Cost of test kits was taken from the national list of prices for medical services issued by the Vietnamese Ministry of Health [31]. Direct medical and direct non-medical costs associated with T2DM treatment for diagnosed T2DM and T2DM complications were obtained from primary data collection in Dong Da hospital—a public provincial level hospital in the Northern Vietnam. The study was approved by the Institutional Review Board (IRB) of the Hanoi University of Public Health, Vietnam on September 16, 2019 (No.438/2019/YTCC-HD3).

Direct medical costs including costs of antidiabetic medicine, examination, lab test, and treatment associated with complications such as cost of hemodialysis for end-stage renal diseases were retrospectively collected from medical records during 2019 using cost-at-charge approach. Individuals with T2DM with or without complications were identified based on the International Classification of Diseases (ICD-10) with the code range from E11 to E14. The IRB of the Hanoi University of Public Health, Vietnam waived the requirement for informed consent. All data were fully anonymized before accessing and analyzing them. A total of 1631 medical records were included in the analysis of direct medical cost.

Direct non-medical costs including food, travel, accommodation, and other relevant non-medical costs of patients and caregivers during treatment were obtained from a prospective survey in 2019. Patients were included in the survey if (1) aged ≥ 18 years; (2) diagnosed with T2DM with or without complications which verified by physicians at Dong Da hospital; (3) willing to participate in the survey. A total of 218 patients were included in the survey for direct non-medical costs as well as utility value. The survey’s objectives as well as risks and benefit were introduced and clearly explained to the patients. Then, they were asked to read and signed informed consent, if they agreed to participate in the survey; otherwise, they would be excluded. Cost of treatment for undiagnosed T2DM patients was estimated by the cost of treatment for diagnosed T2DM multiplied by the ratio between treatment cost of diagnosed versus undiagnosed T2DM previously reported [34]. An annual discount rate of 3% was applied for both costs and outcomes [35]. Costs were estimated in Vietnamese Dong and then converted to US dollars using the exchange rate of $1.00 = 23,143 Vietnamese Dong [36].

#### Utilities

Quality-adjusted life-years (QALYs) were used as health outcomes and the utility scores were derived from primary data collection in the aforementioned hospital. This survey was identical with interview of patient for identifying direct non-medical cost. In short, patients were invited to participate in survey of measuring direct non-medical cost and utility at the same time. A Vietnamese version of the EQ-5D-5L questionnaire was applied to interview patients with permission from the EuroQoL group. Patients were provided the study’s objectives and explained clearly regarding risks and benefit of the questionnaire. Patients were then asked to read and sign the informed consent, if they agreed to participate in this study. The Vietnamese value set was applied to convert answers into utility value [37]. The protocol of the survey was approved by the Review Board of the Hanoi University of Public Health, Vietnam.

### Result presentation

Total costs, life years (LYs), and QALYs for each screening strategy were estimated over lifetime period. To estimate the cost-effectiveness of each screening strategy compared with no screening, an incremental cost-effectiveness ratio (ICER) was calculated and compared with the cost-effectiveness threshold of one gross domestic product (GDP) per capita [38] of Vietnam per QALY gained (US$ 2,715) [39]. Furthermore, the corresponding net monetary benefit (NMB) of all screening strategies was compared to rank their cost-effectiveness. This ranking was also supplemented by incremental analysis (S1 Table). S1 Text provides the Consolidated Health Economic Evaluation Reporting System (CHEERS) checklist.

### Uncertainty analysis

Deterministic one-way sensitivity analysis was performed to demonstrate the robustness of the base case results as well as the significant influence of each parameter on ICER values. Key parameters were varied within the range as follows: transition probabilities (±20%), prevalence of disease (±20%), clinical data (95% Confident Interval (CI)), cost (±20%), and utility (95%CI). The results of univariate sensitivity analysis were presented as tornado diagram. Probabilistic sensitivity analysis was carried out to evaluate parameter uncertainties around the ICER. This was done by the Monte Carlo simulation in which parameters were randomly and simultaneously varied based on their distributions. By randomly sampling from input parameters’ distribution, 1000 estimates for the costs and outcomes of each screening option were generated. The results were exhibited in the cost-effectiveness acceptability curves (CEAC) and cost-effectiveness planes.

## Results

### Cost-utility analysis

One-off screening at CHS and DHC at the age of 40 years onward compared with no screening under a societal perspective yielded the incremental cost of US$ 20.92 and US$ 36.56 and incremental QALYs of 0.01 and 0.02, respectively (Table 2). The ICER for screening at CHS and DHC was US$ 2,077 and US$ 2,139 based on a societal perspective and US$ 539 and US$ 493 based on a provider perspective, respectively. These ICERs were less than the threshold of one GDP per capita in Vietnam (US$ 2,715 per QALY gained), indicating that one-off screening at either CHS or DHC would be cost-effective.

**Table 2:** Cost-effectiveness results of screening options for T2DM in Vietnam at age of 40 years onwards with different screening interval *(*US dollar, 2019*)*.

	Societal perspective			Provider perspective		
	No screening	Screening at CHS	Screening at DHC	No screening	Screening at CHS	Screening at DHC
**Cost of screening**						
One-off screening	0	1.52	3.65	0	0.68	0.37
Annual screening	0	27.46	65.01	0	12.32	6.50
3-yearly screening	0	8.82	20.94	0	3.96	2.09
**Cost of treatment**						
One-off screening	910.70	930.10	943.61	218.1	222.83	226.14
Annual screening	910.70	697.03	750.69	218.1	168.25	181.55
3-yearly screening	910.70	885.81	935.93	218.1	212.90	225.36
**Total cost**						
One-off screening	910.70	931.62	947.26	218.1	223.52	226.51
Annual screening	910.70	724.49	815.71	218.1	180.57	188.05
3-yearly screening	910.70	894.63	856.87	218.1	216.86	227.45
**LYs**						
One-off screening	18.74	18.75	18.76	18.74	18.75	18.76
Annual screening	19.07	19.47	19.51	19.07	19.47	19.51
3-yearly screening	19.07	19.22	19.26	19.07	19.22	19.26
**QALYs**						
One-off screening	17.22	17.23	17.24	17.22	17.23	17.24
Annual screening	17.22	17.62	17.65	17.22	17.62	17.65
3-yearly screening	17.22	17.36	17.39	17.22	17.36	17.39
**Incremental cost**						
One-off screening		20.92	36.56		5.43	8.42
Annual screening		-186.21	-95.00		-37.51	-30.03
3-yearly screening		-16.07	46.17		-1.22	9.37
**Incremental LYs**						
One-off screening		0.01	0.02		0.01	0.02
Annual screening		0.40	0.43		0.40	0.43
3-yearly screening		0.15	0.18		0.15	0.18
**Incremental QALYs**						
One-off screening		0.01	0.02		0.01	0.03
Annual screening		0.40	0.43		0.40	0.43
3-yearly screening		0.14	0.17		0.14	0.17
**ICER (US$/QALY gained)**						
One-off screening		2,077	2,139		539	493
Annual screening		Dominant*	Dominant*		Dominant*	Dominant*
3-yearly screening		Dominant*	268		Dominant*	54
**Incremental NMB** ** **(US$)**						
One-off screening	45,852.2	45,858.5	45,861.8	46,544.8	46,566.5	46,582.6
Annual screening	45,852.2	47,113.7	47,073.3	46,544.8	47,657.6	47,736.9
3-yearly screening	45,852.2	46,253.9	46,375.7	46,544.8	46,931.6	47,005.1

ICER = incremental cost-effectiveness ratio; LYs = life years; QALYs = quality adjusted life years; NMB = net monetary benefit.

*Dominant shows lower costs but higher QALYs.

**At cost-effectiveness threshold of 1 GDP per capita in Vietnam: $ 2,715.3 per QALY gained.

Of all screening options, annual screening at CHS was associated with the lowest lifetime cost at $724.49, but resulted in highest QALYs (17.62). Notably, the annual screening was found to be dominant (i.e., less costly and more effective) compared to no screening under both societal and provider perspectives. Annual screening at CHS and DHC also yielded lower costs (-US$ 186.21 and -US$ 95.00), but higher QALYs (0.40 and 0.43), respectively. Moreover, 3-yearly screening at CHS was also dominant (incremental cost = -US$ 16.07, incremental QALYs = 0.14), while it was cost-effective at DHC (incremental cost = US$ 46.17, incremental QALYs = 0.17) (Table 2).

Based on the incremental NMB results of all screening strategies under a societal perspective compared with no screening, annual screening at CHS was the most cost-effective screening option, followed by annual screening at DHC, 3-yearly screening at DHC, 3-yearly screening at CHS, one-off screening at DHC and one-off screening at CHS (Table 2).

Both annual screening or 3-yearly screening at either CHS or DHC for people aged 30, 35, 40, or 45 years would be cost-effective according to both societal and provider perspectives. In addition, annual screening at either CHS or DHC remained the most cost-effective strategy regardless of starting age to screen in this study (Table 3). Meanwhile, one-off screening was not cost-effective when screening from age of 30 years under a societal perspective.

**Table 3:** Cost-effectiveness results of screening options for T2DM in Vietnam at different starting age (US dollar, 2019).

	Societal perspective			Provider perspective		
	No screening	Screening at CHS	Screening at DHC	No screening	Screening at CHS	Screening at DHC
**Age: 30 years**						
**Total cost**						
One-off screening	1,259.15	1,277.28	1,291.00	309.09	313.74	316.19
Annual screening	1,259.15	936.83	1,035.34	309.09	237.10	243.94
3-yearly screening	1,259.15	1,208.12	1,276.26	309.09	298.87	310.08
**QALYs**						
One-off screening	20.11	20.11	20.12	20.11	20.11	20.12
Annual screening	20.11	20.54	20.57	20.11	20.54	20.57
3-yearly screening	20.11	20.26	20.29	20.11	20.26	20.29
**ICER (US$/QALY gained)**						
One-off screening		2,856	2,957		732	659
Annual screening		Dominant*	Dominant*		Dominant*	Dominant*
3-yearly screening		Dominant*	96		Dominant*	6
**Incremental NMB** ** **(US$)**						
One-off screening	53,337.4	53,335.5	53,335.4	54,287.4	54,299.1	54,310.2
Annual screening	53,337.4	54,832.7	54,804.8	54,287.4	55,532.4	55,596.2
3-yearly screening	53,337.4	53,795.7	53,803.6	54,287.4	54,704.9	54,769.8
**Age: 35 years**						
**Total cost**						
One-off screening	1,082.95	1,102.47	1,117.15	262.73	267.77	270.49
Annual screening	1,082.95	830.86	926.11	262.73	208.73	215.92
3-yearly screening	1,082.95	1,050.27	1,115.84	262.73	257.30	268.28
**QALYs**						
One-off screening	18.74	18.75	18.76	18.74	18.75	18.76
Annual screening	18.74	19.16	19.19	18.74	19.16	19.19
3-yearly screening	18.74	18.89	18.92	18.74	18.89	18.92
**ICER (US$/QALY gained)**						
One-off screening		2,445	2,525		631	573
Annual screening		Dominant*	Dominant*		Dominant*	Dominant*
3-yearly screening		Dominant*	186		Dominant*	31
**Incremental NMB** ** **(US$)**						
One-off screening	49,807.3	49,809.4	49,808.3	50,627.5	50,644.1	50,654.9
Annual screening	49,807.3	51,191.5	51,175.1	50,627.5	51,813.7	51,885.3
3-yearly screening	49,807.3	50,241.7	50,254.9	50,627.5	51,034.7	51,102.5
**Age: 45 years**						
**Total cost**						
One-off screening	747.43	769.64	786.18	176.41	182.21	185.46
Annual screening	747.43	620.62	706.97	176.41	153.38	161.06
3-yearly screening	747.43	745.66	803.78	176.41	178.68	188.72
**QALYs**						
One-off screening	15.56	15.58	15.59	15.56	15.58	15.59
Annual screening	15.56	15.93	15.97	15.56	15.93	15.97
3-yearly screening	15.56	15.70	15.73	15.56	15.70	15.73
**ICER (US$/QALY gained)**						
One-off screening		1,773	1,823		463	426
Annual screening		Dominant*	Dominant*		Dominant*	Dominant*
3-yearly screening		Dominant*	342		17	75
**Incremental NMB** ** **(US$)**						
One-off screening	41,516.2	41,526.6	41.534.5	42,087.2	42,114.0	42,135.2
Annual screening	41,516.2	42,636.8	42.645.5	42,087.2	43,104.0	43,191.4
3-yearly screening	41,516.2	41,876.4	41.907.9	42,087.2	42,443.4	42,522.9

ICER = incremental cost-effectiveness ratio; LYs = life years; QALYs = quality adjusted life years; NMB = net monetary benefit.

*Dominant shows lower costs but higher QALYs.

**At cost-effectiveness threshold of 1 GDP per capita in Vietnam: $ 2,715.3 per QALY gained.

The incremental analysis was performed to confirm the ranking of the most cost-effective screening option (S1 Table). The results confirmed that annual screening at CHS was the most cost-effective in all scenarios of starting age.

### Uncertainty analysis

**Fig 3:**
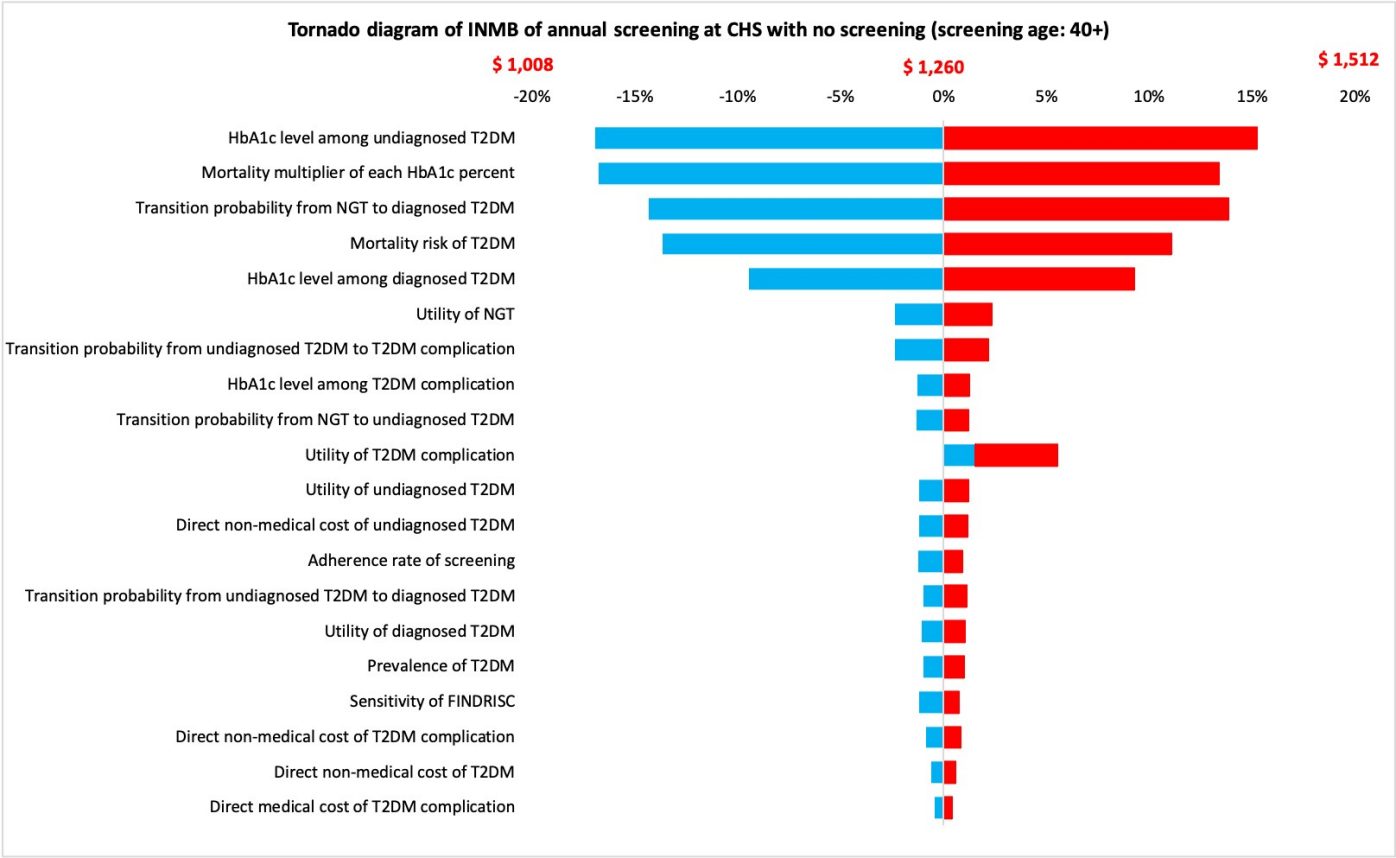
Tornado diagram of incremental net monetary benefit of annual screening at CHS compared with no screening.

Fig 3 illustrates the tornado diagram of the most cost-saving strategy (annual screening at CHS) compared with no screening based on a societal perspective. The incremental NMB was always positive which indicated that annual screening at CHS remained cost-effective at the threshold of one GDP per capita of Vietnam. Furthermore, the incremental NMB was most sensitive to the HbA1c level among undiagnosed T2DM, mortality multiplier of each HbA1c percent increased, transition probability from NGT to T2DM, mortality risk of T2DM, and HbA1c level among diagnosed T2DM.

**Fig 4:**
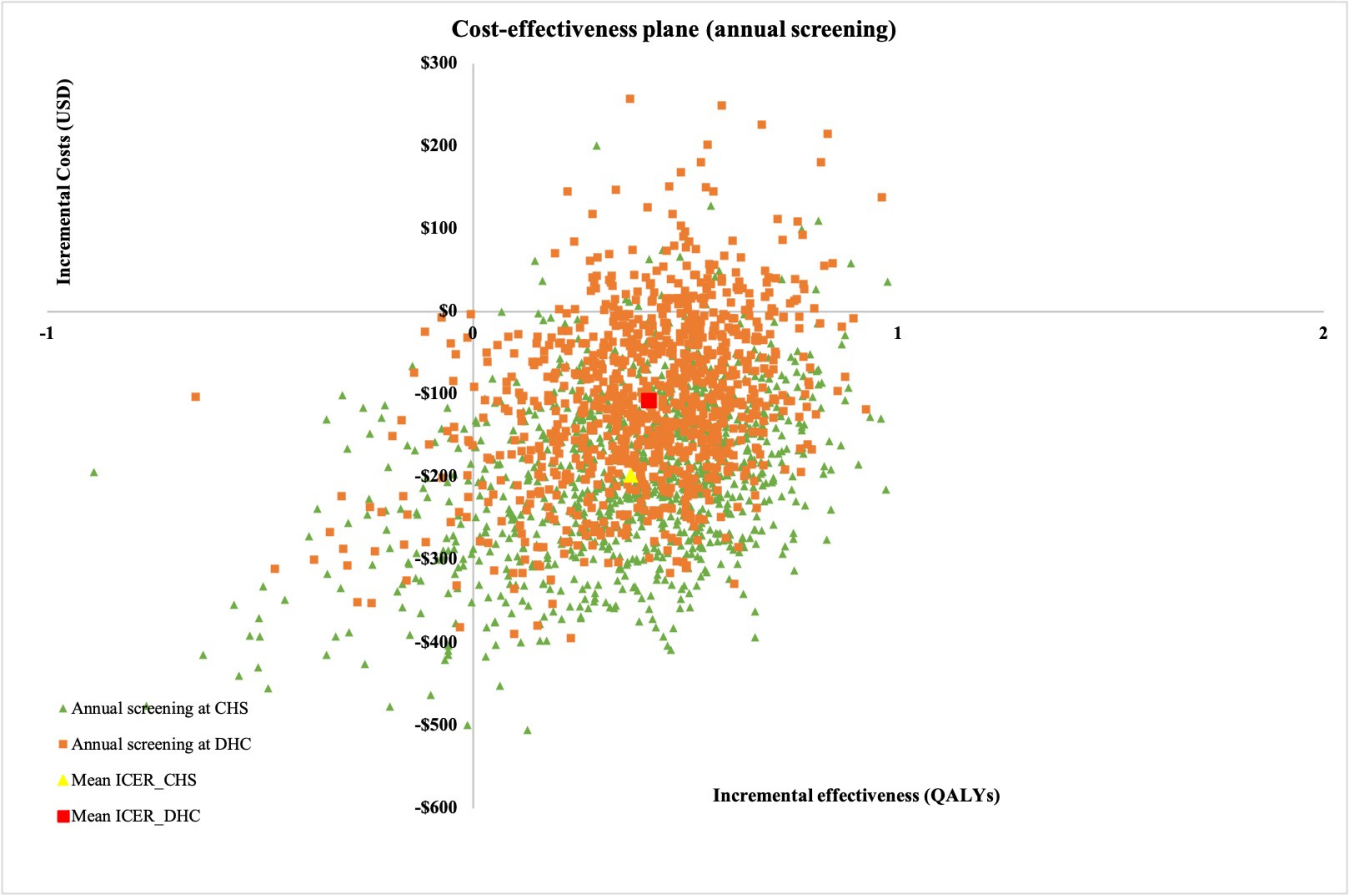
Probabilistic cost-effectiveness plane of screening for T2DM in Vietnam under societal perspective.

**Fig 5:**
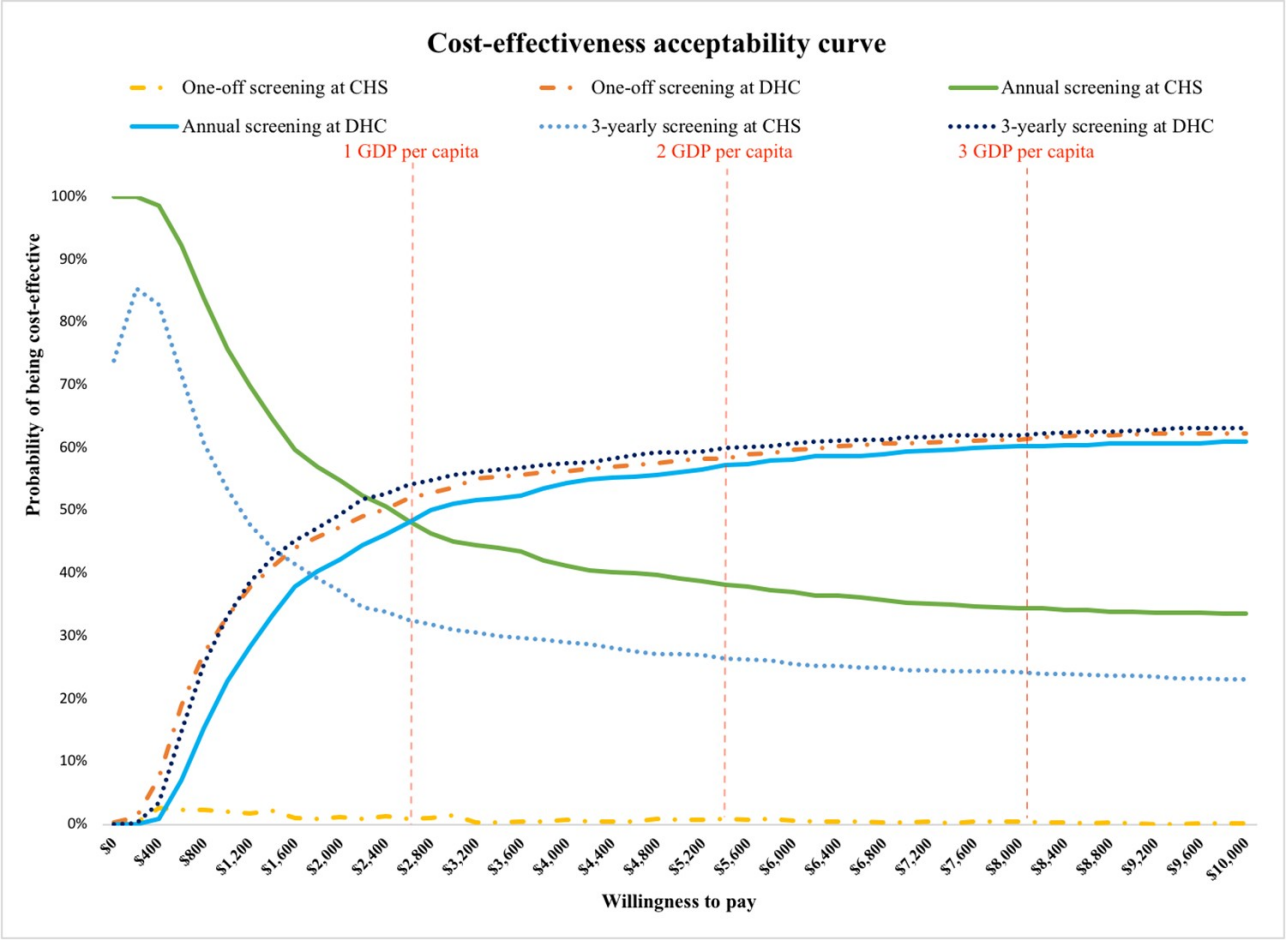
Cost-effectiveness acceptability curve of screening for T2DM in Vietnam under societal perspective.

Based on probabilistic sensitivity analysis results, the cost-effectiveness plane of annual screening (Fig 4) demonstrated that the majority of simulations were in the south-east quadrant. The mean ICER of screening at CHS was below that in screening at DHC, suggesting that this screening option would save more money, while offering almost the same health outcome compared to that in screening at DHC. Moreover, the cost-effectiveness acceptability curve showed that annual screening at CHS had the highest probability of being cost-effective at threshold of one GDP per capita (Fig 5). When the cost-effectiveness threshold was higher than one GDP, annual screening at DHC always remained with a higher probability of being cost-effective. Similarly, 3-yearly screening at CHS had higher probability of being cost-effective compared with 3-yearly screening at DHC at the threshold of one GDP per capita.

## Discussion

Our study aimed to assess the cost-effectiveness of different screening for T2DM strategies in Vietnam. We found that annual screening and 3-yearly screening were cost-effective at the willingness to pay threshold of 1 GDP per capita. Moreover, the incremental analysis showed that annual screening at CHS was the most cost-effective strategy in all scenarios of starting age. Our findings showed that by offering screening, we can save more money by reducing treatment costs, especially treatment costs from disease complications. Therefore, the treatment costs were lower in screening arm compared to no screening arm.

Our study is the first to assess the cost-effectiveness of different screening modalities with different starting ages and screening intervals for T2DM in Vietnam. Our findings were produced by several methods applied in this study. First, we employed opportunistic screening which is currently embedded in current clinical practice in Vietnam. Individuals will be screened, when they come to the health facilities for whatever reasons. This strategy will generally incur less cost of screening program compared to community or mass screening, as it will include the cost of identifying eligible participants, starting-up cost such as advertisement and invitation for screening, and cost of chasing up for non-attenders. This is compliant with the ADA’s recommendation that the screening should be taken place within healthcare settings owing to the need for follow-up and treatment, otherwise individuals with positive tests may not follow the referral or access to further investigation and treatment in community screening [7]. Second, our model is designed in accordance with the healthcare structure in Vietnam, reflecting the real clinical and patient pathway in the country. Recent evidence by Isaranuwatchai et al. highlighted that local context can influence the cost-effectiveness of the intervention in prevention for non-communicable diseases [40]. In this study, we proposed two screening options that follow the healthcare structure in the country, including screening at CHS and DHC. Both CHS and DHC belong to the grassroots level in healthcare system, where almost all primary health care activities are done. Further, they play a major role as a gatekeeper in the system, which is expected to reduce hospital overload in higher levels of health facilities. There are some differences in terms of health accessibility and utilization by the people and consequently associated costs between CHS and DHC. Third, the test was chosen based on availability and acceptance in Vietnamese setting, taking into account the capacity for implementation once the screening program was decided. In reality, in some high-income countries, the HbA1c was chosen for screening in analysis, as it did not require fasting. However, at current capacity in Vietnam, this test is only available in tertiary hospitals and would not be feasible to implement in all healthcare levels country-wide.

The results in our study highlighted that lifetime screening costs at DHC were generally higher than those in CHS under a societal perspective. This is because direct non-medical and time costs associated with screening in DHC were higher than that in CHS, as the distance to DHC especially in rural and mountainous areas is relatively far. Screening at CHS would restrict only patients with positive results to proceed to DHC, therefore costs of transportation and further blood tests can be saved. However, from a provider perspective, screening at CHS was associated with higher costs, because it covers more tests as well as confirmation costs at the district level.

Moreover, our study suggested that at the cost-effectiveness threshold of one GDP per capita in Vietnam, one off screening, annual screening and 3-yearly screening at either CHS or DHC were found to be cost-effective compared to no screening. Our results are in accordance with previous evidence suggesting that screening for T2DM is a cost-effective intervention [14,16,17,41,42]. In particular, studies in countries similar to Vietnamese context indicated favorable results for screening options. For example, the study by Dupka et al. in Bhutan showed that screening for T2DM was cost-effective compared to no screening [41] as well as a study by Rattanavipapong et al. revealed that screening for T2DM, a component of Package of Essential Noncommunicable disease (PEN), was cost-effective in Indonesian settings compared to no screening [43].

However, of all screening intervals in this study, annual screening was the best option which yielded better health outcomes, and saved more money. This could be explained that our study employed opportunity screening rather than universal screening which consumed higher costs of screening and treatment provided to more individuals with diabetes who were detected compared to opportunistic screening. Besides, our screening was carried out among population at-risk of T2DM who were identified by FINDRISC questionnaire. Therefore, annual screening was appropriate with high-risk population following ADA recommendation that more frequent testing could be done depending on initial results and risk status [7]. In addition, our study suggested that one-off screening was not cost-effective when screening for people younger than 35 years old at both CHS and DHC. This is in accordance with the recommendation of Wilson & Jungner (1968) which highlighted that screening should not be once for all [8]. Moreover, in addition to annual screening, our study revealed that 3-yearly screening at either CHS or DHC would also be cost-effective according to both societal and provider perspectives. Consistent with the study of Kahn et al. which applied mathematical modelling found that screening for T2DM between 3–5 years was cost-effective in the US setting [15]. However, the ADA suggested that 3-year interval screening should be carefully considered, as people with false-negative tests must be retested before sufficient time runs out and complications arise [7].

Furthermore, we found that both annual screening and 3-yearly screening for starting age at 30, 35, 40, or 45 years was cost-effective. Similarly, the study in Thailand suggested that screening for T2DM should be taken place for people aged ≥30 [42] as well as the study in the US, recommended that age between 30 and 45 years was appropriate for T2DM screening [15]. Unlike previous evidence in Indonesia, they showed that screening should only be carried out for high-risk groups aged ≥40 [43]. It is noteworthy that in our study only high-risk individuals would be screened as pre-screening by FINDRISC questionnaire was done. Even though there was no explicit method to determine the optimal cut-off for starting age, most studies suggested screening should be performed from age of 40 years. When we considered both screening intervals and screening options, our finding suggested that annual screening at either CHS or DHC for people with starting age ≥ 30 years was the best option, as it yielded highest net monetary benefits. However, budget impact of each starting age scenario should be further investigated for policy decision-making in Vietnam.

Base on uncertainty analysis results, the incremental NMB was most sensitive to the HbA1c level among undiagnosed T2DM, mortality multiplier of each 1% increase in the HbA1c level. However, the incremental NMB was always positive despite parameters varied in plausible range, suggesting that these parameters did not affect the cost-effectiveness conclusion. Nevertheless, future studies may be further investigated on predicting the HbA1c level among undiagnosed T2DM.

Several limitations in our study needs to be addressed. First, the outcomes of screening program in our study would be underestimated, as we did not consider screening for prediabetes in this study. In reality, patients would go through prediabetes state before progression to T2DM. By lowering the cut-off points of screening test, the prediabetes could be detected and the effects from intervention would reduce the risk of developing T2DM and T2DM complications consequently. However, the shortage of evidence in terms of prevalence and incidence of prediabetes in Vietnam makes this work impossible. Second, our results slightly underestimated the indirect effect of screening on the benefit of total population as a whole. In reality, participants with negative results could be aware of their risk and receive the recommendation from clinicians. Accordingly, they might modify their lifestyle and consequently reduce the risk of developing T2DM. Future studies may investigate measuring these indirect benefits of screening programs. Third, our model simulated a hypothetical cohort of individuals who were free of comorbidities and complications. While the comorbidities and complications would be presented during the initial (i.e., first few years) of any screening program, it was less likely the case when the screening program had been implemented for a period of time. Next, due to the lack of local data such as the incidence of T2DM, some parameters were obtained from neighboring countries. Lack of longitudinal data in the country regarding individuals with diabetes also leads to the impossibility of model calibration to fit with actual data. Besides, we considered model parameters were independent. However, ideally the potential correlation between parameters should be explored. Different scenarios on starting age were evaluated in our study, though the age was set arbitrarily. Future studies may explore the optimal cut-off point for age that should be started for screening for T2DM. Currently, Vietnam has no country-specific cost-effectiveness threshold, the actual threshold may change the conclusion in some scenarios in our study. The need of establishing a country-specific threshold that would be done systematically and applied scientific methods would be warranted.

## Conclusion

Screening for T2DM is cost-effective compared to no screening, current practice in Vietnam. Annual screening at CHS was found to be the best screening strategy for high-risk population. Meanwhile, one-off screening was only cost-effective in the Vietnamese setting when screening for people aged ≥ 35 years. The Vietnamese government should consider allocating resources for T2DM screening in the country. T2DM screening should be included in the Vietnamese health benefits package and annual screening at either CHS or DHC should be implemented.

## Supporting information

S1 Table Incremental analysis of different T2DM screening strategy in Vietnam under societal perspective (US dollar 2019). (DOCX)

S1 Text CHEERS (Consolidated Health Economic Evaluation Reporting System) checklist. (DOCX)
