# Supplementary material for: Economic evaluation of population-based type 2 diabetes mellitus screening at different healthcare settings in Vietnam
Source: PLoS One. 2021 Dec 23;16(12):e0261231. doi: 10.1371/journal.pone.0261231 (PMC8700026; doi:10.1371/journal.pone.0261231)
Supplement: S1 Table — (DOCX) [file pone.0261231.s001.docx]

**Supplement S1 - Economic evaluation of population-based type 2 diabetes mellitus screening at different healthcare settings in Vietnam**

Phung Lam Toi, Olivia Wu, Montarat Thavorncharoensap, Varalak Srinonprasert, Thunyarat Anothaisintawee, Ammarin Thakkinstian, PhD, Nguyen Khanh Phuong, Usa Chaikledkaew

S1 Table. Incremental analysis of different T2DM screening strategy in Vietnam under societal perspective (US dollar 2019).

| **Screening option** | **Total cost** | **Total QALYs** | **Incremental Cost*** | **Incremental QALYs*** | **ICER** |
| --- | --- | --- | --- | --- | --- |
| **Screening from age of 30+** |  |  |  |  |  |
| Annual screening at CHS | 936.8 | 20.539 | - | - |  |
| Annual screening at DHC | 1,035.3 | 20.565 | 98.5 | 0.026 | 3,789.1 |
| 3-yearly screening at CHS | 1,208.1 | 20.257 | 172.8 | -0.308 | Dominated |
| No screening | 1,259.1 | 20.107 | 223.8 | -0.458 | Dominated |
| 3-yearly screening at DHC | 1,276.3 | 20.285 | 240.9 | -0.28 | Dominated |
| One-off screening at CHS | 1,277.3 | 20.113 | 241.9 | -0.452 | Dominated |
| One-off screening at DHC | 1,291.0 | 20.118 | 255.7 | -0.447 | Dominated |
| **Screening from age of 35+** |  |  |  |  |  |
| Annual screening at CHS | 830.9 | 19.159 | - | - |  |
| Annual screening at DHC | 926.1 | 19.188 | 95.3 | 0.029 | 3,285 |
| 3-yearly screening at CHS | 1,050.3 | 18.889 | 124.2 | -0.299 | Dominated |
| No screening | 1,082.9 | 18.742 | 156.8 | -0.446 | Dominated |
| One-off screening at CHS | 1,102.5 | 18.75 | 176.4 | -0.438 | Dominated |
| 3-yearly screening at DHC | 1,115.8 | 18.919 | 189.7 | -0.269 | Dominated |
| One-off screening at DHC | 1,117.2 | 18.755 | 191.0 | -0.433 | Dominated |
| **Screening from age 40+** |  |  |  |  |  |
| Annual screening at CHS | 724.5 | 17.618 | - | - | - |
| Annual screening at DHC | 815.7 | 17.65 | 91.2 | 0.032 | 2,851 |
| 3-yearly screening at CHS | 894.6 | 17.364 | 78.9 | -0.286 | Dominated |
| No screening | 910.7 | 17.222 | 95.0 | -0.428 | Dominated |
| One-off screening at CHS | 931.6 | 17.232 | 115.9 | -0.418 | Dominated |
| One-off screening at DHC | 947.3 | 17.239 | 131.6 | -0.411 | Dominated |
| 3-yearly screening at DHC | 956.9 | 17.395 | 141.2 | -0.255 | Dominated |
| **Screening from age of 45+** |  |  |  |  |  |
| Annual screening at CHS | 620.6 | 15.931 | - | - | - |
| Annual screening at DHC | 707.0 | 15.966 | 86.3 | 0.035 | 2,467 |
| 3-yearly screening at CHS | 745.7 | 15.697 | 38.7 | -0.269 | Dominated |
| No screening | 747.4 | 15.565 | 40.5 | -0.401 | Dominated |
| One-off screening at CHS | 769.6 | 15.577 | 62.7 | -0.389 | Dominated |
| One-off screening at DHC | 786.2 | 15.586 | 79.2 | -0.38 | Dominated |
| 3-yearly screening at DHC | 803.8 | 15.73 | 96.8 | -0.236 | Dominated |

*(*) Incremental costs and QALYs were calculated after ruling out the strongly dominated alternatives*
